# Quality Attributes of Cupuaçu Juice in Response to Treatment with Crude Enzyme Extract Produced by *Aspergillus japonicus* 586

**DOI:** 10.4061/2011/494813

**Published:** 2011-11-01

**Authors:** Maria Francisca Simas Teixeira, Jerusa Souza Andrade, Ormezinda Celeste Cristo Fernandes, Nelson Durán, José Luiz de Lima Filho

**Affiliations:** ^1^Laboratório de Microbiologia, Universidade Federal do Amazonas, 69077-000 Manaus, AM, Brazil; ^2^Departamento de Tecnologia de Alimentos, Instituto Nacional de Pesquisas da Amazônia (INPA), Caixa Postal 478, 69060-001 Manaus, AM, Brazil; ^3^Coordenação de Pesquisa, Universidade Nilton Lins, 69058-030 Manaus, AM, Brazil; ^4^ILMD, FIOCRUZ, 69057-070 Manaus, AM, Brazil; ^5^Laboratório de Físico-Química Orgânica, Instituto de Química, Universidade Estadual de Campinas, 13084-971 Campinas, SP, Brazil; ^6^Departamento de Bioquímica, Universidade Federal de Pernambuco, 50670-420 Recife, PE, Brazil

## Abstract

Cupuaçu (*Theobroma grandiflorum* Schum) is an Amazonian Basin native fruit whose fruit pulp is consumed as a juice which presents high density, viscosity, and turbidity. Pectic enzymes, usually yielded by microorganisms, are used to reduce the juice viscosity and turbidity. The present study aims to evaluate the use of pectic enzymes when processing cupuaçu juice. The cupuaçu juice was obtained by using *Aspergillus japonicus* 586 crude enzyme extract and incubation at 50°C with agitation (140 rpm) for one hour. Enzyme activities were determined, and the juices were evaluated as to their yield, turbidity, viscosity, and chemical composition. The juice produced by using crude enzyme extract presented higher soluble solids, reducing sugars, and lower viscosity and turbidity.

## 1. Introduction

Commercial preparations of pectic enzymes used in food industries are frequently derived from filamentous fungi [1], mainly strains of *Aspergillus niger* [2–4]. Species of this group include fungi with asexual reproduction (deuteromycete), included in the class Hyphomycetes, which are characterized by morphological structures typical of the genus *Aspergillus*. In the Amazon, *Aspergillus japonicus* 586, *niger* group, are preserved in the mycology collection (collection of microorganisms) of the Universidade Federal do Amazonas. They are thermotolerant species, isolated from various natural substrates, such as wood shavings and parts (peel, pulp and seeds) and kept in a liquid medium with pH 2–5 [5].

In recent decades, the highlight of deuteomycetes is associated with the production of bioactive compounds, such as enzymes (cellulases, pectinases, and proteases), which present many advantages for being recognized as safe (GRAS). Highlighted by the production of biocatalysts, fungal species are used in food industry and in bioremediation processes, contributing, respectively, to lower the residue rate and biodegradation need [6, 7].

Pectinases have application in the food industry to degrade pectin. These enzymes are used in the fruit juice extraction process on account of facilitating the filtration, increasing yield, and reducing the viscosity and turbidity caused by pectin [8–10]. Among the pectic enzymes, polygalacturonase and pectinesterase bring about pectin depolymerization and de-esterification, respectively. Traditionally, crude preparations containing several pectinases are used by fruit juice and alcoholic beverage industries, although some processes require only a few of them [6].

The Amazon presents high fruit species diversity, some of them are already part of traditional crops [10], yet most still come from natural extractivism. Some are known worldwide, but most are only known and consumed locally. Some fruits whose chemical composition is known arouse great interest due to the presence of bioactive substances. Other fruits are preferred by their very attractive flavor [11, 12]. Cupuaçu is a native fruit which is cultivated in the Amazon [13, 14]. Its pulp has high consumer acceptance and is used for the preparation of juices, ice creams, candies, cream, jam, dessert, and liquors. Due to the consumer's preference within and outside the Amazon, the cupuaçu is a fruit that stands out as the most promising for industrialization. Thus, the development of studies can help to encourage industrialization and, especially, to give value to the Amazonian biodiversity.

The pulp of cupuaçu presents a pseudoplastic behavior [15]. Among the traditional juices, the cupuaçu is preferred by consumers in the Amazon. This juice is thick and viscous and has high concentrations of colloids and solids particles. These features cause problems for the industrialization of the juice. The flow of juice inside the tubular system of industrial equipment is complicated, and the physical stability of the manufactured product is low.

The present study is undertaken so as to couch the pulp industrialization, the increase on the demand for cupuaçu nectars, and the importance of microbial enzymes for improving the process and especially the Amazonian biodiversity valorization. This research aims to evaluate the effect of crude extract of enzymes produced from *Aspergillus japonicus* 586 on the cupuaçu juice physicochemical characteristics.

## 2. Material and Methods

### 2.1. Microorganism

In this study, *Aspergillus japonicus* 586 was selected randomly from the collection (Culture Collection DPUA) maintained by the Federal University of Amazonas (Universidade Federal do Amazonas (UFAM)), located in Manaus, Amazonas, Brazil. The stock culture was obtained in test tubes (148 × 230 mm) containing Czapek yeast extract agar (CYA). The cultures were kept for seven days at 30°C, and their viability was monitored every 24 hours [16].

### 2.2. Crude Enzyme Extract

A suspension of 5 × 10^6^ spores/mL of liquid medium containing 0.5% (w/v) citric pectin was obtained (aseptically) from the stock culture in CYA. The pectinolytic enzymes were produced in the culture medium containing (g/L of distilled water) KH_2_PO_4_ (2.0), NH_4_SO_4_ (1.0), MgSO_4_·7H_2_O (0.1), Na_2_HPO_4_·7H_2_O (0.9), yeast extract (1.0), and citric pectin (5.0), and the pH was adjusted to 3.0 with a dilute sulfuric acid solution. The medium was sterilized during 15 min at 120°C. After autoclaving, a spore suspension plus 5 mL of polidimetilsilorano (1% w/v) aqueous solution was added to 5 L of liquid culture medium to give a final concentration of 5 × 10^6^ spores/mL. The fermentation was carried out in Bioflo III fermenter (New Brunswick Scientific, USA) containing 5,000 mL of liquid culture medium using the following conditions: temperature of 28°C, shaking of 140 rpm, and aeration rate of 0.6 v/v/min (volume of air/volume medium/minute) during 120 hours. The crude enzyme extract was recovered by vacuum filtration through a 0.45 mm membrane [5, 17].

### 2.3. Cupuaçu Pulp

Mature fruits were collected from crops cultivated in the vicinity of Manaus, Amazonas, Brazil. The fruits were washed, and the shell was broken. The pulp was the separated from the seed by hand with the aid of scissors and immediately used in the experiments.

### 2.4. Treatments

In the experiment four juice samples (in duplicate) were obtained by varying the liquid phase (crude enzyme extract or water) and process (incubated and not incubated). To obtain the juice quantities of 100 g of pulp and 100 mL of water or crude enzyme extract were used. The trituration was done for three minutes in a blender (high speed). The incubation was conducted using 300 mL beakers (opened), Dubnoff shaker digital model NT 232, with shaking of 140 rpm, temperature 50°C for one hour. Enzyme activity was interrupted by heat treatment (immersion immediately after trituration to control without incubation and after incubation for the others) for five minutes in boiling water bath and cool immediately in ice water bath. Filtration (under manual pressure) was done with gauze tissue (folded eight times) and with quantitative recovery of juice and residue.

### 2.5. Physicochemical Characteristics

The volume (mL) of juice was measured, and the residue (filtration under compression) was weighed after drying (constant weight) in an oven at 80°C. The pH was determined by pH meter (Procyon-10) and acidity (expressed as percentage of citric acid) by titration with 0.1 M NaOH (phenolphthalein as indicator). Ascorbic acid was extracted with 0.5% oxalic acid and measured by titration with 2,6-dichlorophenolindophenol [18]. The sugars were extracted with water and quantified by the Somogyi-Nelson [19]. Reducing sugars were determined prior to and total sugars following heated-acid hydrolysis. The nonreducing sugars were determined by the difference found between total and reducing sugars. On account of presenting very high absorbance (1.49 to 2.81) it was necessary to dilute the juice (10 times) with water so as to perform the turbidity analysis. To evaluate the turbidity, the volume of 5 mL of juice was diluted into 50 mL of distilled water and the absorbance read on a Perkin-Elmer spectrophotometer at 660 nm [20]. The findings were expressed as diluted-juice absorbance units. The viscosity was determined by Ostwald viscometer reading [21]. 

### 2.6. Endopolygalacturonase Activity

Endopolygalacturonase activity was measured viscosimetrically by the Tuttobello and Mill method [21] by using 250 *μ*L of the samples and 5.75 mL of 0.2% (w/v) citric pectin in 0.025 M acetate buffer (pH 5.0) containing 1 mM ethylenediaminetetraacetic acid (EDTA). The blank was prepared with 250 *μ*L of buffer solution. The incubation was conducted at 50°C during 10 and 60 minutes for the crude enzyme extract and juices, respectively, and then cooled in an ice water bath. The readings were made on the same Ostwald viscometer. Viscosimetric unit (U) was defined as the enzyme quantity required to decrease the initial viscosity by 50%. The enzymatic activity was expressed as U/mL/min.

### 2.7. Exopolygalacturonase Activity

Exopolygalacturonase activity was determined using the 3,5-dinitrosalicylic acid (DNS) reagent [22]. The reactive mixture contained 250 *μ*L of 0.5% (w/v) citric pectin in 0.025 M acetate buffer (pH 5.0), 1 mM ethylenediaminetetraacetic acid (EDTA), and 250 *μ*L of the crude enzymatic extract or 500 *μ*L of the juice. The incubation time was 10 (crude enzymatic extract) and 60 (juices) minutes at 50°C. After incubation, 500 *μ*L of DNS solution was added to each tube and boiled for 5 minutes. After cooling, 5 mL of distilled water was added. The absorbance at 575 nm was measured. One enzymatic unit (U) was defined as the enzyme quantity that releases one *μ*mol of reducing group per minute under the assay conditions. The enzymatic activity was expressed in nmol/mL/min. 

### 2.8. Pectinesterase Activity

Pectinesterase activity was evaluated by the pH decrease of the medium and by titration of carboxylic groups released according to the modified methods [23, 24]. For the activity analysis the volumes of 2 mL of 1% (w/v) citric pectin solution in 0.025 M Tris-acetate buffer (pH 6.5) and 1 mL of crude enzyme extract or 3 mL of juice were used. The blank was prepared in the same way except that the samples were substituted by the buffer solution. After incubation for 2 hours at 50°C the reaction was stopped in a boiling water bath for 3 minutes. The samples were cooled in an ice bath and titrated with 0.1 M NaOH solution. A pectinesterase unit (U) was defined as the quantity of enzyme which liberates a microequivalent of carboxylic group in one hour of reaction under the described conditions. The results were expressed as U/mL/min.

### 2.9. Amylase and Cellulase Activity

To detect the presence of amylase and cellulase a solid medium was used and each 8 mm diameter “cup-plate” was completed with 100 *μ*L of the samples. The Petri plate was protected by aluminum paper enclosing it (so as to prevent evaporation) and incubated at 37°C for 18 hours. Following incubation and addition of 0.1 N iodine solution (amylases) or 0.1% Congo red (cellulases) positive reaction was detected by a translucent halo display (measured in cm) around each “cup-plate.” Enzyme extracts from *Aspergillus awamori* (amylase positive) and celluzyme (Novo Nordisk) were used as controls for amylase and cellulase, respectively [5, 25].

## 3. Results and Discussion

### 3.1. Enzymatic Activities

**Figure 1:**
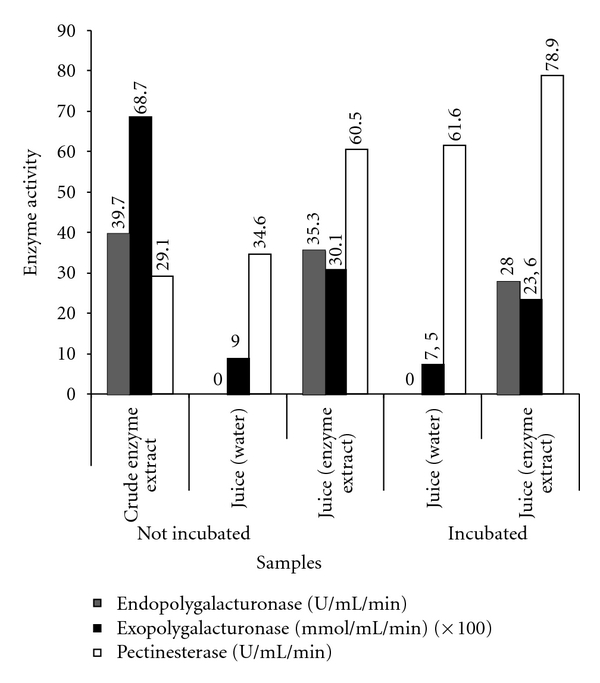
Pectinase activities in the crude enzyme extract produced by *Aspergillus japonicus* 586 and juices of cupuaçu (*Theobroma grandiflorum* Schum).

Differences in the activities of pectinases were detected in the crude enzyme extract produced by *Aspergillus japonicus* 586, as well as in the cupuaçu juices (Figure 1). The pectic enzymes (pectinesterase and polygalacturonases) were detected in crude extract from *Aspergillus japonicus* 586, and their activities were affected by singled or combined carbon source different concentrations [5]. Exopolygalacturonase activity was also detected in the juice obtained without the addition of crude enzyme extract. The incubation decreased the activity of exopolygalacturonase. On the other hand, incubation during one hour at a temperature of 50°C increased the activity of pectinesterase, which showed higher values, especially in the juice from the crude enzyme extract. The activity of pectinesterase detected in juices was higher compared to crude enzyme extract.

Positive reaction (90 mm halo) to the cellulase was detected in the crude enzyme extract from *Aspergillus japonicus* 586. Amylase was neither in the juice nor in crude enzyme extract. Qualitative detection of cellulase may also indicate the possibility of reducing agroindustrial residue and thus minimizing the negative effect on the environment, when being used in large quantities needed by major agroindustries.

Commercial products of pectic enzymes, containing pectinesterase, polygalacturonase, and pectin lyase, have been used in wine and juice industries to increase the yield and improve pigment, flavor, transmittance, and viscosity quality [6]. On account of there being no commercial enzyme available at the time the present experiment was carried out we were obliged to use the traditional method of making cupuaçu juice by using water.

### 3.2. Yield, Viscosity, and Turbidity

**Figure 2:**
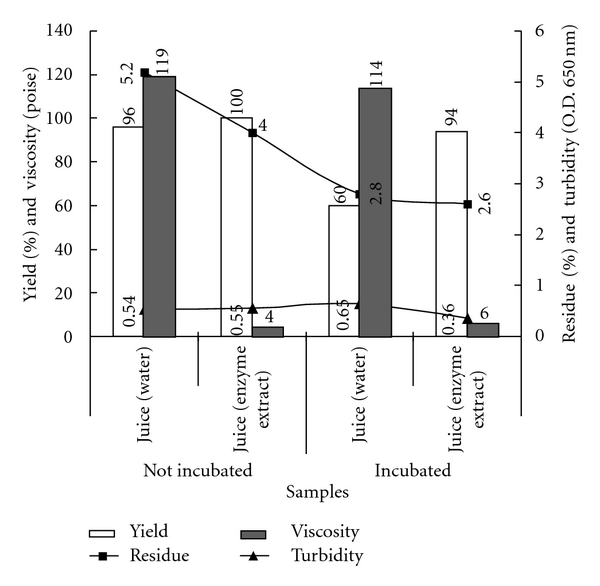
Effect of crude enzyme extract produced by *Aspergillus japonicus* 586 on the yield and characteristics of juices of cupuaçu (*Theobroma grandiflorum* Schum).

The major aim of this study is to evaluate the use of enzymes extracted from a fungus isolated in Brazilian Amazon for processing the juice from a native plant consumed by local people. In this research, the beneficial effect of crude enzyme extract from *Aspergillus japonicus* 586 is indicated by the results shown in Figure 2. In addition to increasing the juice yield, the amount of waste to be discharged was diminished. As the agroindustrial waste is polluting, the smallest volume to be discharged into the environment implies a lesser need for bioremediation processes. With the exception of juice extracted with water and subjected to incubation, the others showed high yield in the range from 94 to 100%. Pulp yield increase and waste reduction were also achieved with the use of commercial pectinase when obtaining cupuaçu pulp [26].

Produced with the ratio of 1 : 1 (pulp : liquid) and subjected to filtration (with manual pressing), the juice still presented high turbidity and had to be diluted so as turbidity could be assessed. The incubation of the juice obtained with water caused an increase in turbidity and a remarkable decrease in viscosity. However, during incubation the enzymes present in crude enzyme extract caused a significant reduction in the turbidity especially in the viscosity in the cupuaçu juice. The results also show that the incubation process was essential to enzyme activity since optimum temperature and time lapse are essential for the activity of any enzyme.

The results shown in Figure 2 indicated that the incubation process is essential for the action of enzymes, reducing viscosity and lowering turbidity and, therefore, altering the characteristics of the final product. This is the most interesting result, confirming the usefulness of this technique for the quality control analysis and improving the industrial process.

### 3.3. Chemical Composition

The effect of the enzymes alone or in combination with incubation is shown by the data presented in Table 1. Taking just incubation into account the juice obtained with the crude enzyme extract shown to be sweeter. This characteristic is indicated by the lowest values of pH and Brix/acidity ratio and the highest acidity. Moreover, in degradation of pectin by the action of pectinesterase, there occurs the release of methanol and H^+^. Thus, this ion also contributes to the decrease in pH [6].

However, incubation for one hour on recipient (beaker) open and under shaking (140 rpm) at 50°C resulted in loss of volatile acids, with more intensity in the juice obtained with water (3.67 mg/min) than in the one with enzyme extract (2.0 mg/min). The incubation also caused oxidation of ascorbic acid, and as this vitamin is more stable in acidic medium [18], the loss in the juices (more acidic) obtained with crude enzyme extract was lower. The conversion of sugars by hydrolysis is also favored by heating and acidity [19]. The occurrence of such conversion during incubation is indicated by the increase and decrease of reducing and nonreducing sugars, respectively.

The methanol production was not evaluated. However, information about toxicological aspects is necessary since this handmade method could be replaced by industrial process. The methanol production is positively associated with enzyme activities of pectinesterase and pectate lyase. Several studies have found that methanol can be produced from the hydrolysis of methyl ester groups in pectins by endogenous pectinesterases and occurs naturally at a low level in fresh squeezed juices, stored and canned fruit juices, and most of the alcoholic beverages. The addition of pectic enzymes can cause an increase in methanol level during mashing, fermentation, and aging stages fruit wines and distilled fruit spirits [27–29].

As a consequence, this process probably intensifies the release of methanol and increases the risk for consumers. Methanol is toxic to human through ingestion and inhalation. Accidental intake of the compound will result in severe intoxication due to accumulation of toxic metabolites. In the body, methanol is metabolized in the liver and converted first to formaldehyde and then to formate. Acute intoxication of methanol usually causes headache, vertigo, fatigue, nausea, vomiting, blurred vision, blindness, and even death [30–32].

The presence and amount of methanol were not determined in this study. The incubation was conducted for one hour at 50°C in open beaker with stirring. Later, the juices were subjected to heat treatment for five minutes in boiling water, also in open beakers. Methanol is a volatile compound (the boiling point is 65°C), and its absence in the controlled cashew apples wine may be due to its loss by evaporation during pasteurization [33]. To avoid potential health hazard, the analysis of this methanol content in the fresh juice or in the processed beverage is desirable because the equipments for industrial process are closed. It is important to note that the methanol is an undesirable product in juice processing and is a common problem in the industrial beverages production.

In the Amazon, cupuaçu pulp is frozen and stored under freezing. At the time of consumption, this pulp is added with water and sugar to get the juice. Currently this handmade process of obtaining juice tends to be replaced by the industrial process [31, 32]. Thus, the use of pectic enzymes produced by microorganisms, and especially selected in the Amazon, is a suggestion to improve the process and also obtain the desirable characteristics [9, 33] of the processed product. The results shown in Figure 2 indicated that the incubation process is essential for the action of enzymes, reducing viscosity and lowering turbidity and agroindustrial residues, therefore altering the characteristics of the final product. This is the most interesting result, confirming the usefulness of this technique for the quality control analysis and to improve the industrial process.

## 4. Conclusion

The reductions in viscosity, turbidity, and the amount of waste are the major positive contribution of the use of crude enzymatic extract yielded by *Aspergillus japonicus *586 for the production of juice of cupuaçu. Also, it stands out as a positive contribution to efforts to encourage future research in the Amazon, concerning the isolation and selection of microorganisms, and specially investigating the use of these processing enzymes in sound and acceptable products from this and other fruits, which make up the Amazonian biodiversity. The joint efforts, consequently, will contribute to sustainable development and extractivism Amazonian biodiversity.

## Figures and Tables

**Table 1:** Effect of crude enzyme extract produced by *Aspergillus japonicus* 586 on the chemical composition of juices cupuaçu (*Theobroma grandiflorum* Schum).

Juice characteristics	Not incubated		Incubated	
	Juice (water)	Juice (enzyme extract)	Juice (water)	Juice (enzyme extract)
pH	3.0	2.9	3.0	2.9
Titratable acidity (%)	0.92 ± 0.02	1.04 ± 0.02	0.70 ± 0.01	0.92 ± 0.01
Ascorbic acid (mg%)	13.87 ± 0.32	14.11 ± 0.31	5.44 ± 0.20	8.1 ± 0.02
Soluble solids (°Brix)	6.6 ± 0.13	6.6 ± 0.13	6.8 ± 0.13	7.1 ± 0.14
Brix/acidity ratio	7.17 ± 0.01	6.35 ± 0.09	9.71 ± 0.20	7.72 ± 0.33
Reducing sugars (%)	0.49 ± 0.03	0.58 ± 0.03	0.61 ± 0.01	1.09 ± 0.08
Nonreducing sugars (%)	1.83 ± 0.01	1.73 ± 0.01	1.34 ± 0.22	1.05 ± 0.13
